# Case report: A case of Culler-Jones syndrome caused by a novel mutation of *GLI2* gene and literature review

**DOI:** 10.3389/fendo.2023.1133492

**Published:** 2023-03-03

**Authors:** Yiwen Zhang, Bingzi Dong, Yu Xue, Yunyang Wang, Jing Yan, Lili Xu

**Affiliations:** ^1^ Department of Endocrinology, Affiliated Hospital of Qingdao University, Qingdao, China; ^2^ Department of Gastroenterology, Affiliated Hospital of Qingdao University, Qingdao, China

**Keywords:** Culler-Jones Syndrome, *GLI2* gene, hypogonadotropic hypogonadism, anosmia, high- throughput sequencing

## Abstract

Culler-Jones syndrome is a rare clinical phenomenon with diverse manifestations and is prone to misdiagnosis. We report one patient who presented with a 10-year history of anosmia and a 1-year history of epididymal pain. Kallmann syndrome was suspected initially. The results of his laboratory tests, imaging, and genetic testing, however, combined to provide a conclusive diagnosis of Culler-Jones syndrome. With the aid of high-throughput sequencing technology, the *GLI2* gene c.527A>G (p.Tyr176Cys) heterozygous mutation in the child was identified. No published works have yet described this mutation site. We described Culler-Jones syndrome in a child at length. We recommend that Culler-Jones syndrome be taken into account when considering the spectrum of disorders associated with abnormal growth and development in children. Once diagnosed, individualized hormone replacement treatment is required for each patient.

## Introduction

Culler-Jones Syndrome (CJS, OMIM615849) is an autosomal dominant genetic disorder caused by mutations in the *GLI2* gene located on the long arm of chromosome 2 at position q14. Its clinical phenotype indicates significant heterogeneity and is primarily characterized by hypopituitarism with or without postaxial polydactyly, facial dysmorphism, and intellectual disability (1). Kallmann Syndrome (KS, OMIM147950), also known as olfactory deficiency-hypogonadism syndrome, has an overlap of causative genes and clinical phenotypes with CJS (2, 3), and needs to be distinguished in the diagnosis. We present a case of CJS with the primary symptoms being hypogonadotropic hypogonadism, growth retardation, and anosmia. We broaden the spectrum of diseases associated with abnormal growth and development in adolescents and enhance clinicians’ understanding of the disease in the hopes of assisting with the early diagnosis and treatment of the disease by summarizing the clinical manifestations, genetic testing of the patient, and reviewing the pertinent literature.

## Case presentation

### General information

The 15-year-old child came to our hospital in December 2021 with a 10-year history of anosmia and a 1-year history of epididymal pain. Ten years prior, the boy was discovered to have lost his ability to smell without nasal congestion or a runny nose. Even after receiving anti-inflammatory, anti-allergic, and nerve feeding therapies during multiple visits to the otolaryngology department, his conditions continued. One year ago, the child developed pain in the epididymis. Epididymitis was identified after numerous visits to the andrology and urology departments. Anti-inflammatory and anti-allergic medication was provided to him, but the symptoms did not considerably relieve. The patient was the second child of healthy non-consanguineous parents, with a birth weight of 2850g. Although the Apgar score was unknown, he was born in reportedly good condition. His mother denied any history of specific diseases or specific medications during pregnancy. He was breastfed exclusively from birth to 7 months, at which point complementary foods were introduced. There was no distinction between him and his peers when it came to head raising, sitting, crawling, walking, teething, and talking. At six months, he increased in height by 4 cm. There was no family history of genetic disorders. On physical examination: he was found to be clear-headed and cognitively capable, with a BMI of 26.03 kg/m^2^. He was 164cm tall (20th centile) and weighed 70kg (85th centile). He had sparse hair, no special facial features, and loss the sense of smell in both noses (the patient was asked to close his eyes and test his left nose, right nose and both noses with water, soap and white vinegar, respectively; the patient failed to name the odor correctly). He had no beard, no enlarged laryngeal nodes or voice change, no significant abnormalities on cardiopulmonary and abdominal examination. Several purple stripes were visible on his bilateral thighs. He had sparse pubic hair (Tanner II stage), a childlike penis that was about 2 cm long, and testicles that were around 2 ml in volume on each side.

### Laboratory and imaging test

**Figure 1:**
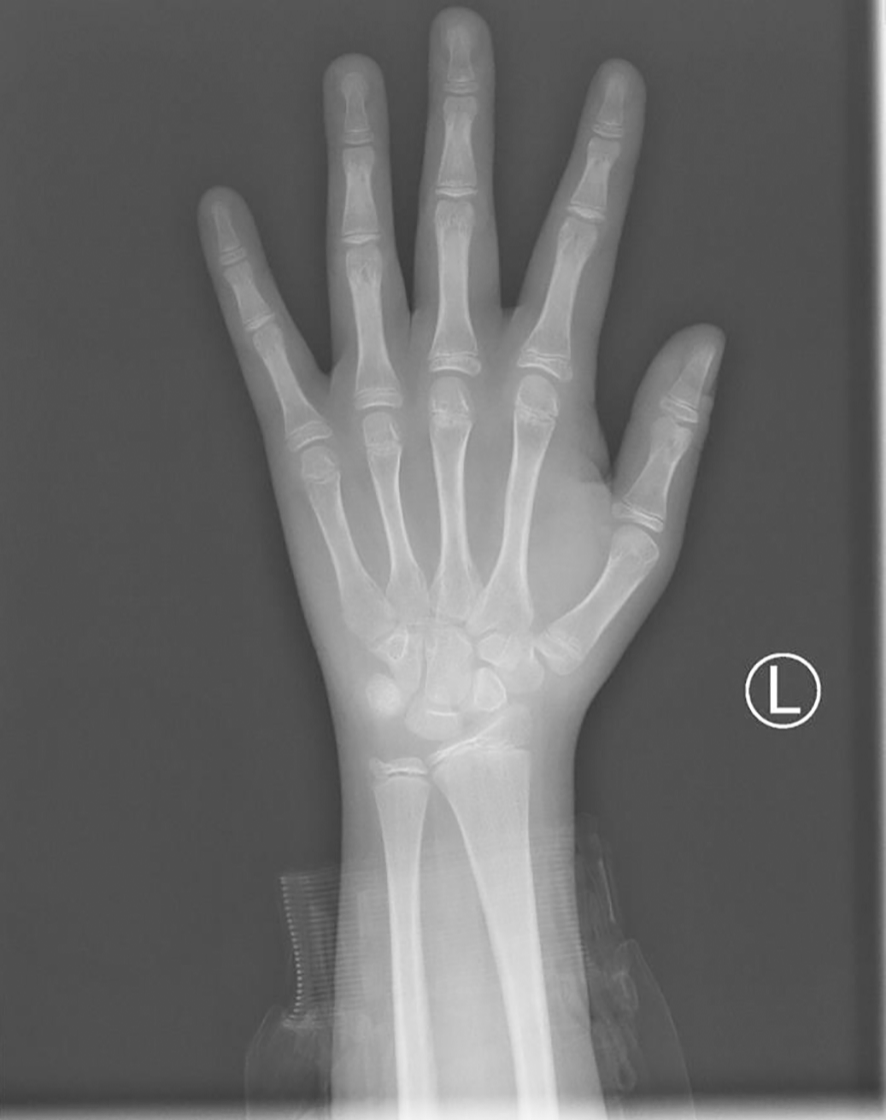
The bone age radiograph of the patient’s left wrist.

**Figure 2:**
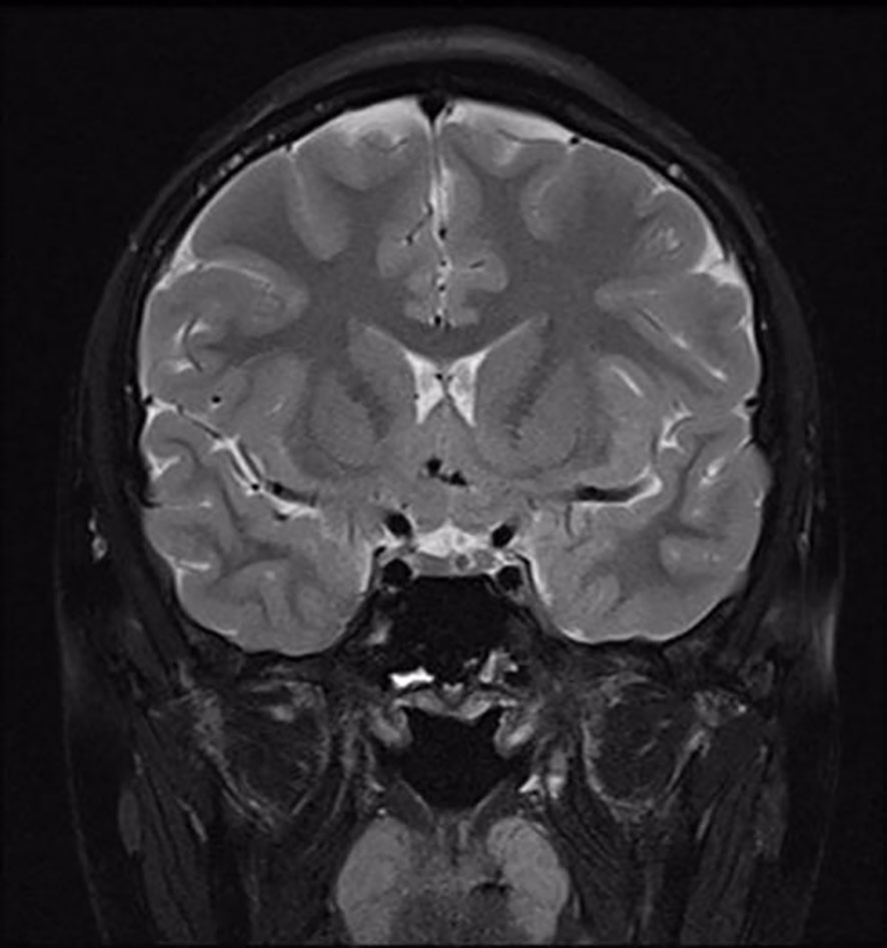
MRI image of the pituitary gland. A nodule in the lower left pituitary is observed.

The clonidine-induced (300ug, oral administration) growth hormone (GH) stimulation test was performed and showed a peak GH of 7.83ng/ml. Also, the insulin-induced(10IU, intravenous injection)hypoglycemic growth hormone stimulation test showed a peak GH of 3.42ng/ml and a simultaneous blood glucose minimum of 2.1mmol/l (**Table 1**). We also performed other endocrine-related tests: insulin-like growth factor-1(IGF-1) 281ng/mL (reference range for children of the same age and sex in China (4), 282.86-930.74 ng/mL), follicle-stimulating hormone(FSH) 1.06mIU/mL, luteinizing hormone(LH) 0.57 mIU/mL, prolactin 159.30 mIU/mL, testosterone 0.24nmol/L, estradiol 63.60pmol/L, progesterone<0.16nmol/L, GnRH(Triptorelin 100ug, subcutaneous injection)stimulation test: LH(0-30min-60min-120min) 0.8-7.95-8.14-6.89 mIU/mL, circadian cortisol rhythm: cortisol(8am-4pm-0am)173.0-93.8-21.2nmol/L, ACTH(8am-4pm-0am)9.96-13.1-32pg/mL. Thyroid function was within reference range. The results related to bone metabolism markers are as follows: serum calcium 2.42mmol/L (reference range 2.11-2.52 mmol/L), serum phosphorus 1.71mmol/L(reference range 0.85-1.51 mmol/L), parathyroid hormone(PTH) 45.1pg/mL (reference range 15-65 pg/mL), 25OHD 13.6ng/mL, ALP225U/L(reference range 45-125 U/L), N-MID 85.90 ng/mL (reference range 6.87-29.05 ng/mL), β-CTX2.20 ng/mL (refer-ence range 0.131-0.9 ng/mL), PINP405 ng/mL (reference range 21.32-112.8 ng/mL). Routine blood, urine and stool tests, liver and kidney function, electrolytes, blood coagulation, infectious markers, blood lipids, and tumor markers were generally normal. A dual-energy X-ray absorptiometry (DXA) scan was performed and this revealed a lumbar spine BMD of 0.707g/cm^2^ (Z score -2.5), total hip BMD of 0.769 g/cm^2^ (Z score -1.3), and femoral neck BMD of 0.738g/cm^2^ (Z score -1.4). The patient’s bone age radiograph showed that there are already eight left carpal bones, which corresponds to a bone age of approximately 12.5 years according to the standard bone age atlas (**Figure 1**). Right testicle dimensions were estimated by the reproductive system ultrasonography to be 1.6 x 1.0 x 0.8 cm, and left testicle dimensions to be 1.5 x 1.1 x 0.6 cm. On color Doppler ultrasound examination, his breast development was detected. Dynamic enhanced pituitary MRI indicated a 4×3mm nodule in the lower left pituitary, indicating a high possibility of microadenoma (**Figure 2**).

**Table 1:** the results of GH stimulation test.

	0min	30min	60min	90min	120min
Clonidine-induced GH stimulation test (ng/ml)	3.20	0.54	0.22	4.50	7.83
Insulin-induced GH stimulation test(ng/ml)	1.71	3.42	0.79	0.83	1.12
Simultaneous blood glucose(mmol/L)	4.30	2.10	4.80	4.20	4.30

### Genetic testing

**Figure 3:**
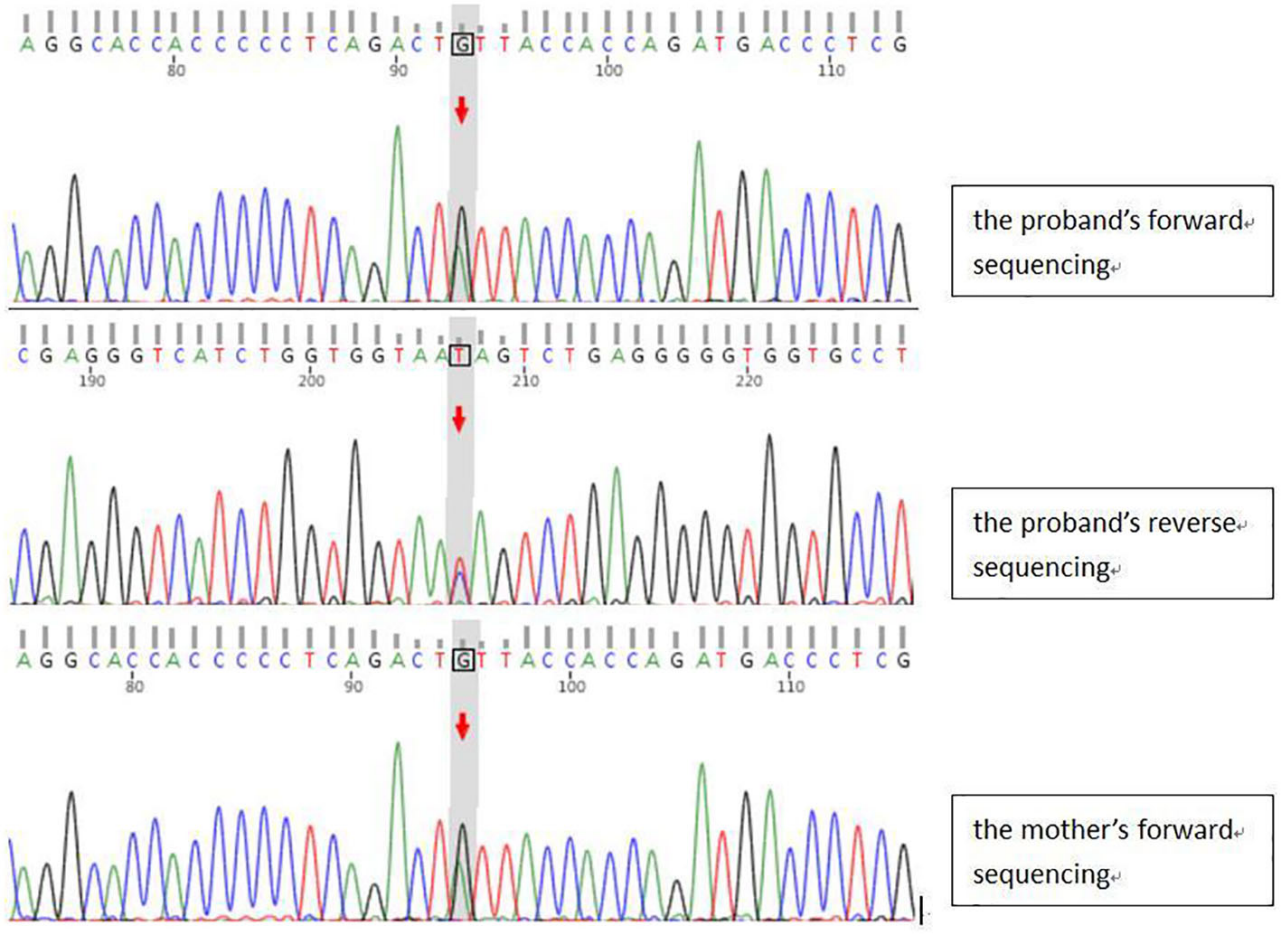
The peak map of gene sequencing.

With the informed consent signed by the guardian of the child, 2ml of peripheral venous blood samples of the child and parents were extracted, and EDTA was added for anticoagulation. A genomic library was constructed. Sequencing reactions were performed on the Illumina platform, and the GATK software package was used to analyze the sequencing data. The sequenced fragments were aligned with the UCSC hg reference genome by BWA, and finally the mutations were interpreted for pathogenicity with reference to the Standards and guidelines for the interpretation of sequence variants published by ACMG. Eventually, the *GLI2* gene c.527A>G (p.Tyr176Cys) heterozygous mutation was detected, and the same mutation was detected in the patient’s mother, which was a missense mutation in the coding region of the *GLI2* gene, resulting in a change of the amino acid tyrosine to cysteine at position 176 of the encoded protein (**Figure 3**). The effect of the mutation on GLI2 protein function was moderate, as assessed by Mutation Assessor software. The Mutation Taster software was used to analyze the pathogenicity of the variant, and the results suggested that the variant was a possible pathogenic variant.

### Diagnosis

The child was presented with 10 years of anosmia and 1 year of epididymal pain. In addition, he was discovered to be delayed in height, with a childish penis, sparse pubic hair and undersized testicles. Auxiliary examination revealed his partial deficiency of growth hormone, hypogonadotropic hypogonadism and delayed bone age. With the aid of high-throughput sequencing technology, the *GLI2* gene c.527A>G (p.Tyr176Cys) heterozygous mutation in the child was identified. In addition to the aforementioned, the patient was identified as having Culler-Jones syndrome.

### Treatment and follow-up

In this case, the patient was young and had no fertility requirement currently. Therefore, both testosterone replacement treatment and combination intramuscular injection of human chorionic gonadotropin (HCG) and human menopausal gonadotropin (HMG) are options. In combination with the views of the patient and family, testosterone undecanoate capsules 40 mg qd were temporarily administered as replacement therapy. However, sex hormone replacement therapy may accelerate epiphyseal closure, and the child already had significant delayed bone age and partial growth hormone deficiency, so there is an indication for exogenous growth hormone supplementation. Considering the possibility that exogenous growth hormone supplementation may promote pituitary tumor growth, we decided not to supplement it for the time being after communicating with the family, and to closely monitor the height and bone age changes of the child. For the secondary low bone mass, alfacalcidol soft capsule 0.25 UG qd and elemental calcium 600mg qd were given. After 3 months of treatment, we followed up the child, who had grown 2 cm taller in that time but had not significantly developed pubic hair, testicles, or penis, nor had his serum testosterone level increased. It is suggested that the child is insensitive to the testosterone replacement treatment. Combined with the previous history of epididymal pain, the possibility of a concurrent primary gonadal abnormality in the child cannot be excluded.

## Discussion

In 1984, Culler and Jones already described the characteristics of CJS (5): postaxial polydactyly, multiple pituitary hormone deficiencies, dysmorphic facial features and autosomal dominant pattern of inheritance. In recent years, CJS has been reported in the United States, Germany, Italy, Brazil, and China (6–8), and its clinical phenotype has been widely broadened. Patients with *GLI2* mutations have been reported with growth retardation, ectopic posterior pituitary, postaxial polydactyly, epilepsy, cleft lip and palate, hypoglycemia, atrioventricular septal defect, and congenital urethral stenosis (9, 10), and some heterozygous mutations of *GLI2* also exist in the gene database of normal people. The heterogeneity and diversity of clinical phenotypes in this disease are associated with incomplete penetrance of gene mutations (11). Mutations in the *GLI2* gene alone may not be sufficient to mediate the generation of abnormal phenotypes and must act in conjunction with other genes and the environment, which reflects the influence of the environment on gene expression.

Located at position q14 on the long arm of chromosome 2, *GLI2* is a large and polymorphic gene containing 13 exons and encoding a protein containing 1,586 amino acids. GLI2 protein is a transcription factor containing a zinc finger DNA binding domain with transcriptional repressive activity at the amino terminus and transcriptional activating activity at the carboxyl terminus. Under the regulation of different species and cellular environments, this protein plays a role in promoting or repressing transcription, which regulates the expression of target genes in the Hedgehog (Hh) pathway together with *GLI1*, *GLI3* and *Shh (* 12). The Hh pathway plays a crucial role in the development of central nervous system, distal limbs and the occurrence and development of various tumors. In humans, numerous congenital deformities and disorders (13), including Greig syndrome and holoprosencephaly (HPE, OMIM: 236100), are associated with abnormalities in the Hh pathway.

It has been found that mutations in the *GLI2* gene not only causes CJS, but also may lead to HPE and KS. CJS and HPE can be considered as two ends of a continuous phenotypic spectrum of the same disease (2), with the milder phenotype being CJS and the more severe phenotype being diagnosed as HPE. CJS,HPE and KS have a certain degree of overlap in both mutant genes and clinical phenotypes (2, 3). KS is a genetically heterogeneous disease involving multiple genes, and *KAL1* is the most common causative gene identified so far (14), while *CHD7*,*WDR11*,*PROKR2*,*FEZF1*,*SE-MA3A*,*SOX10*,*HS6ST1*,*NEL* and *GLI2* have also been reported. The main clinical manifestations of KS are hypogonadotropic hypogonadism and anosmia. Besides these two manifestations, CJS is often combined with a variety of pituitary hormone deficiency, especially growth hormone deficiency, and *GLI2* is the most common causative gene identified so far. This patient started with anosmia, accompanied by hypogonadotropic hypogonadism. KS was suspected initially, but the thyroid hormone, adrenocorticotropic hormone and growth hormone in KS patients were usually within the normal range, while the patient in this case was combined with growth hormone deficiency, and whole exon gene sequencing indicated a heterozygous missense mutation in the *GLI2* gene c.527A>G in the child with a change in tyrosine to cysteine at position 176. In conclusion, the clinical characteristics and molecular phenotype of this child were more consistent with Culler-Jones syndrome, and the final diagnosis was Culler-Jones syndrome. In terms of treatment, there are three main regimens for idiopathic hypogonadotropic hypogonadism in men (15, 16): pulsatile gonadotropin-releasing hormone (GnRH) treatment, combined intramuscular injection of HCG and HMG as well as testosterone replacement therapy. Considering that the mutation of *GLI2* gene can cause primary hypopituitarism, subcutaneous pulse infusion of GnRH is inappropriate for the patient. Combined intramuscular injection of HCG and HMG can promote testicular development, testosterone secretion and spermatogenesis, which may eventually contribute to the recovery of fertility. Testosterone replacement therapy can promote the development of secondary sex characteristics, but cannot reverse infertility. Long-term oral administration of testosterone can cause erythrocytosis, acne, prostatic hyperplasia, premature epiphyseal closure and many other problems. This patient was young and had no fertility requirement for the time being. Therefore, both testosterone replacement treatment and combination intramuscular injection of human chorionic gonadotropin (HCG) and human menopausal gonadotropin (HMG) are options. In combination with the views of the patient and family, testosterone undecanoate capsules 40 mg qd were temporarily given. In the meanwhile, intensive monitoring of height and bone age changes in the child is also critical. The treatment can be adjusted to combined HCG/HMG intramuscular injection when the patient has fertility requirements.

In conclusion, we report a case of a child with anosmia, partial GH deficiency, hypogonadotropic hypogonadism and secondary low bone mass, in which high-throughput gene sequencing suggested a missense mutation in *GLI2* gene c.527A>G (p.Tyr176Cys). This mutation site has not yet been documented in any published works. The Mutation Taster software interpreted it as a possible pathogenic variant, nevertheless, there is a lack of functional prediction of this variant based on animal models, and more in-depth basic studies are expected to verify the relationship between this variant and the occurrence of CJS in the future. Reviewing the relevant literature, mutations in the *GLI2* gene usually induce hypoglycemia, hypopituitarism and abnormal neurological development. In the future, we will continue to follow up the child’s blood glucose level, growth and development status, and the levels of various pituitary hormones. Culler-Jones syndrome is a rare clinical phenomenon with diverse manifestations and is prone to misdiagnosis. Genetic testing is a powerful tool to assist in diagnosis. We anticipate that this case report will expand the spectrum of diseases related to adolescent growth and development, improve clinicians’ understanding of Culler-Jones syndrome, and enhance their capacity to identify, diagnose and treat it.

## Data availability statement

The data presented in the study are deposited in the SRA database, accession number: PRJNA937021. Temporary Submission ID: SUB 12889215.

## Ethics statement

Written informed consent was obtained from the minor(s)’ legal guardian/next of kin for the publication of any potentially identifiable images or data included in this article.

## Author contributions

YZ synthesized the patient’s information and conducted the literature review to write this report. BD performed the framework and clarified information during the editing process. YX and YW gave the constructive discussions to the article. JY contributed to the English grammar. LX was the principal mentor for the project and revised important intellectual content. All authors contributed to the article and approved the submitted version.
